# Genome-Wide Identification and Bioinformatics Analysis of the FK506 Binding Protein Family in Rice

**DOI:** 10.3390/genes15070902

**Published:** 2024-07-10

**Authors:** Fanhao Nie, Minghao Wang, Linlin Liu, Xuefei Ma, Juan Zhao

**Affiliations:** The Key Laboratory for Quality Improvement of Agricultural Products of Zhejiang Province, College of Advanced Agricultural Sciences, Zhejiang A&F University, Hangzhou 311300, China; 15802977913@163.com (F.N.); 18205435123@163.com (M.W.);

**Keywords:** rice, FKBP, abiotic stress, gene structure, motif

## Abstract

The FK506 Binding Protein (FKBP), ubiquitously present across diverse species, is characterized by its evolutionarily conserved FK506 binding domain (FKBd). In plants, evidence suggests that this gene family plays integral roles in regulating growth, development, and responses to environmental stresses. Notably, research on the identification and functionality of *FKBP* genes in rice remains limited. Therefore, this study utilized bioinformatic tools to identify 30 *FKBP*-encoding genes in rice. It provides a detailed analysis of their chromosomal locations, evolutionary relationships with the *Arabidopsis thaliana* FKBP family, and gene structures. Further analysis of the promoter elements of these rice *FKBP* genes revealed a high presence of stress-responsive elements. Quantitative PCR assays under drought and heat stress conditions demonstrated that genes *OsFKBP15-2*, *OsFKBP15-3*, *OsFKBP16-3*, *OsFKBP18*, and *OsFKBP42b* are inducible by these adverse conditions. These findings suggest a significant role for the rice *FKBP* gene family in stress adaptation. This research establishes a critical foundation for deeper explorations of the functional roles of the *OsFKBP* genes in rice.

## 1. Introduction

FK506 Binding Proteins (FKBPs) are a class of cellular receptor proteins, widely present in organisms and categorized as immunophilins, which are capable of binding with immunosuppressive drugs such as Cyclosporine-A (CsA), FK506, and rapamycin [1]. Since the 1980s, concerted efforts have enabled the successful isolation and purification of various FKBPs which specifically bind to FK506 and rapamycin. These interactions between FKBPs and immunosuppressants have revealed the significant potential of immunophilins in organ transplantation applications [2,3].

All members of the FKBP family contain an FK506 binding domain (FKBd), composed of approximately 110 amino acids. This region serves not only as the binding site for FK506 and rapamycin but also as the active site for peptidyl-prolyl cis-trans isomerase, resulting in a highly conserved amino acid sequence and tertiary structure for this domain [4,5,6]. In the plant FKBP family, molecular weights of different members vary due to diverse structural compositions. For example, *Arabidopsis* AtFKBP12 and riceOsFKBP12 are composed solely of an FKBd domain and have a molecular weight of only 12 kDa, whereas rice OsFKBP75 and wheat wFKBP77 possess multiple domains and exceed a molecular weight of 70 kDa [7]. Studies have also shown that in plants and other species, larger FKBP molecules include additional structural domains beyond the FKBd, such as Tetratricopeptide Repeat (TPR) and calmodulin-binding domains [8,9]. For instance, *Arabidopsis* AtFKBP62 and AtFKBP65 can bind to the heat shock protein HSP90 through their TPR domain, thus regulating *Arabidopsis*’sheat tolerance [8,9,10,11]. Research by Meiri [9,12] indicates that AtFKBP62 and AtFKBP65 influence the production of small heat shock proteins related to high-temperature stress repair, yet they exhibit opposite functions during prolonged high-temperature stress developmental processes in *Arabidopsis*.

Studies have highlighted the critical roles of *FKBP* gene family members in *Arabidopsis* growth, development, and stress responses. For instance, the *Arabidopsis* mutant *fkbp42* of AtFKBP42 displays spiral root growth and an overall slow development, thus also known as TWD1 (TWISTED DWARF1). Further research has shown that TWD1 interacts with ATP Binding Cassette B auxin efflux transporter-associated proteins AtABCB1, AtABCB4, and AtABCB19, potentially affecting the cellular localization of these transport proteins and thereby disrupting root auxin transport, impacting plant growth and development [13]. *Arabidopsis* AtFKBP72 is involved in the biosynthesis of Very Long-Chain Fatty Acids (VLCFAs); the disruption of its function interrupts VLCFA production, leading to abnormal cell division and resulting in the *Arabidopsis* mutant with the “PASTICCINO” phenotype [8]. AtFKBP15-1 and AtFKBP5-2 inhibited lateral root development in *Arabidopsis* [10]. In rice, OsFKBP20-1a and OsFKBP20-1b share 85% homology and are both expressed in the nucleus. Although their expression patterns differ across tissues, both are induced by high temperatures and drought. According to Ahn, *OsFKBP20-1a* is highly expressed in various plant tissues and is induced by high temperatures and drought stress [14]. In contrast, the expression of *OsFKBP20-1b* begins to rise 24 h post-stress and is also present in the cytoplasm. OsFKBP20-1a can bind with SUMO (Small Ubiquitin like Modifier)-conjugated enzymes, and through SUMOylation, these enzymes can bind with other target proteins, thus participating in the high-temperature stress response; however, their specific functions and mechanisms of action have yet to be elucidated [15].

In wheat, *FKBP* genes are unevenly distributed across all 21 chromosomes, with their subcellular localization primarily in the nucleus, endoplasmic reticulum, cytoplasm, and chloroplasts [16]. The *FKBP* gene family has also been identified in cucumbers, with 19 members predicted to be expressed in chloroplasts, the nucleus, cytoplasm, cytoskeleton, and peroxisomes [17]. The *FKBP* gene family not only plays a vital role in the life processes of plants but it is also indispensable in animals. In studies on the Asian migratory locust (*Locusta migratoria* L.), researchers have identified 10 genes containing the FKBP_C (peptidyl-prolyl cis-trans isomerase) domain, which may play significant roles in embryo development and immune processes [18].

As a critical food crop and model plant, rice contains multiple genes encoding FKBPs, yet research on the identification and functionality of this gene family in rice is limited. This study, through bioinformatics methods, identified 30 genes encoding FKBP in rice (*Oryza sativa* L.) and analyzed their chromosomal locations, evolutionary relationships with the *Arabidopsis* family, gene structures, and promoter elements. This foundational study lays the groundwork for in-depth functional research on this family and the molecular breeding of rice for stress tolerance.

## 2. Materials and Methods

### 2.1. Identification of FKBP Genes

Rice genome sequences were retrieved from the Phytozome database (https://phytozome-next.jgi.doe.gov/ (accessed on 9 May 2024), and amino acid sequences of *Arabidopsis thaliana AtFKBP* genes were sourced from the TAIR database (https://www.arabidopsis.org/index.jsp (accessed on 9 May 2024)) to serve as reference sequences. Local BLAST searches using TBtools were conducted to identify potential members of the rice OsFKBP gene family. The characteristic FKBP_C conserved domain (PF00254) associated with the *FKBP* gene family was obtained as a Hidden Markov Model from the InterPro database (https://www.ebi.ac.uk/interpro/entry/pfam/ (accessed on 10 May 2024)), and searches were performed using HMMER3.0 software to identify candidate members [19,20]. These candidate members were then merged, and a reverse BLASTp analysis was conducted using the NCBI (National Center for Biotechnology) database (https://blast.ncbi.nlm.nih.gov/Blast.cgi (accessed on 11 May 2024)) to refine the selection of *OsFKBP* family genes further [21]. Domain predictions and verifications were carried out using the NCBI CD-search tool (https://www.ncbi.nlm.nih.gov/Structure/bwrpsb/bwrpsb.cgi (accessed on 11 May 2024)), with any candidates missing domains or exhibiting redundant transcripts excluded from the final *OsFKBP* gene list.

### 2.2. Phylogenetic Analysis of FKBP Genes

Amino acid sequences of rice *OsFKBP* and *A. thaliana AtFKBP* genes were aligned using MAFFT v7.490 software employing the E-INS-i strategy. Phylogenetic trees were constructed using the Maximum Likelihood (ML) method with FastTree v2.1.11 software, employing the Jones-Taylor-Thornton (JTT) protein evolution model. Trees were visualized using FigTree v1.4.3 software [22,23].

### 2.3. Chromosomal Localization and Collinearity Analysis of FKBP Genes

The chromosomal localization of the *OsFKBP* family genes was visualized using TBtools v2.041 software based on annotated rice genome information. Collinearity analysis of rice *OsFKBP* genes and between rice and *Arabidopsis FKBP* genes was conducted using the One Step MCScanX-Super Fast component of TBtools v2.041 software [24].

### 2.4. Analysis of FKBP Gene Structures

Structural information for rice *OsFKBP* genes was extracted using TBtools v2.041 software for gene structure analysis. The MEME suite (https://meme-suite.org/meme/tools/meme (accessed on 12 May 2024)) was utilized to analyze the conserved motifs within the OsFKBP sequences, with the motif count was set to 10 and all other parameters were at default settings. Structural domain predictions were conducted using the NCBI-CDD database (https://www.ncbi.nlm.nih.gov/Structure/bwrpsb/bwrpsb.cgi (accessed on 12 May 2024)) [25]. The visualization of gene structures, conserved motifs, and structural domains was performed using TBtools v2.041 software.

### 2.5. Analysis of Gene Promoter Cis-Acting Elements

Potential promoter sequences located 2000 bp upstream from the ATG start codon of rice *OsFKBP* genes were extracted using TBtools v2.041 software. These sequences were analyzed for promoter *cis*-acting elements using the PlantCARE database (https://bioinformatics.psb.ugent.be/webtools/plantcare/html/ (accessed on 12 May 2024)) and visualized using TBtools v2.041 software [26].

### 2.6. Experimental Materials and Conditions

The experimental subject was the *Japonica* rice variety Nipponbare. Seedlings were cultured hydroponically: seeds were soaked until germination. After germination, seedlings were maintained in a rice nutrient solution [27]. At 14 days post-germination, seedlings underwent stress treatment with 22% PEG 6000 and were subjected to a temperature of 45 °C under a light/dark cycle of 16/8 h with a light intensity of 200 mmol m^−2^ s^−1^ for 24 h. Aerial parts of the rice (including stems and leaves) were sampled at 0, 6, 12, and 24 h post-treatment and immediately frozen in liquid nitrogen.

### 2.7. Total RNA Extraction, Reverse Transcription, and Quantitative RT-PCR

Total RNA was extracted using the TRIzol method. Reverse transcription was performed using reverse transcription kits provided by Yeasen Biotech (Shanghai, China) Co., Ltd., and quantitative RT-PCR was conducted using Yeasen’s Hieff^®^ qPCR SYBR Green Master Mix (11201ES08, Shanghai, China). *Actin* was used as the internal reference gene for expression analysis, conducted using a BIO RAD real-time fluorescence quantitative PCR instrument. The qPCR reaction volume was 10 μL, consisting of 5 μL of Hieff^®^ qPCR SYBR Green Master Mix (Shanghai, China), 0.2 μL each of forward and reverse primers (10 μM), 2 μL of cDNA template, and 2.6 μL of RNase-free H_2_O. The qPCR protocol included an initial denaturation at 95 °C for 5 min, followed by 40 cycles of denaturation at 95 °C for 10 s, annealing at 55–60 °C for 20 s, and extension at 72 °C for 20 s. The reaction was performed with biological triplicates as described previously [28,29]. Relative gene expression was calculated using the formula: Relative Expression = 2^−ΔΔCT^. Primer sequences used for RT-PCR are listed in Table S2.

## 3. Results

### 3.1. Identification and Evolutionary Analysis of FKBP Genes

**Figure 1:**
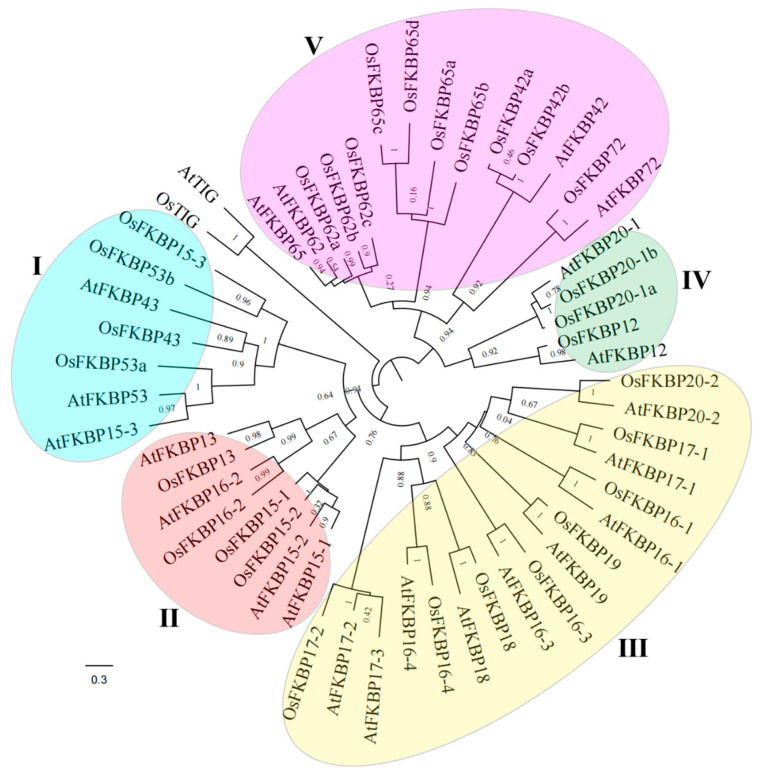
Phylogenetic Tree of FKBPs in rice and *Arabidopsis*. The phylogenetic tree, constructed using FastTree v2.1.11 software, summarized the evolutionary relationships between 53 members of the OsFKBP and AtFKBP families, and named rice OsFKBP family members based on the similarity of the AtFKBP family in *Arabidopsis*. The tree displays five phylogenetic subfamilies (numbered I to V and marked with different alternating colors). The OsFKBP family in rice was one where internal nodes may not necessarily provide true phylogenetic relationships between different offspring.

Utilizing both BLAST and HMM search strategies, we identified thirty members of the OsFKBP family within the rice genome. These proteins were named sequentially from OsTIG, and OsFKBP12 through to OsFKBP72, based on their homology to the *A. thaliana* FKBP family, with corresponding accession numbers provided in Table S1. To elucidate the evolutionary relationships of FKBPs across multiple species, we performed sequence alignments and constructed a phylogenetic tree of FKBP family proteins from both rice and *Arabidopsis* (Figure 1). The phylogenetic analysis revealed that, with the exception of the distantly related TIG proteins, the remaining FKBPs from rice and *Arabidopsis* segregate into five distinct subgroups. Subgroup I comprises OsFKBP15-3, OsFKBP43, OsFKBP53a/b from rice, and AtFKBP15-3, AtFKBP43, and AtFKBP53 from *Arabidopsis*. Subgroup II includes OsFKBP13, OsFKBP15-1/2, OsFKBP16-2 from rice, alongside AtFKBP13, AtFKBP15-1/2, and AtFKBP16-2 from *Arabidopsis*. Subgroup III contains OsFKBP16-1/3/4, OsFKBP17-1/2, OsFKBP18, OsFKBP19, OsFKBP20-2 from rice, and AtFKBP16-1/3/4, AtFKBP17-1/2/3, AtFKBP18, AtFKBP19, and AtFKBP20-2 from *Arabidopsis*. Subgroup IV consists of OsFKBP12, OsFKBP20-1a/b from rice, and AtFKBP12 and AtFKBP20-1 from *Arabidopsis*. Finally, subgroup V includes OsFKBP42a/b, OsFKBP62a/b/c, OsFKBP65a/b/c/d, OsFKBP72 from rice, and AtFKBP42, OsFKBP62, OsFKBP65, and OsFKBP72 from *Arabidopsis*.

### 3.2. Chromosomal Localization Analysis of FKBP Genes

**Figure 2:**
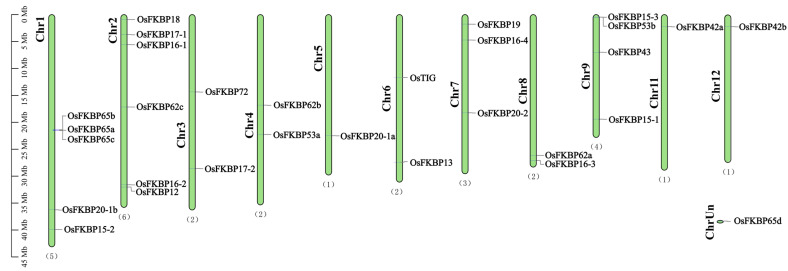
Localization of *OsFKBP* genes in rice on chromosomes. Using TBtools, 30 rice *OsFKBP* genes were mapped onto chromosomes, with each gene’s position on the chromosome represented by a line, and the total number of *FKBP* genes on each chromosome represented by parentheses at the top of each chromosome. This ratio is measured in megabytes (Mb)*. OsFKBP65d* is not anchored to a chromosome. ChrUn, unknown in which chromosome. (1)–(6), the number of identified FKBP distributed on this chromosome.

Utilizing the annotation data from the rice genome, 30 rice *OsFKBP* genes were mapped to chromosomes using the TBtools software. A chromosomal distribution map was created for these genes (Figure 2). The localization analysis reveals that, with the exception of chromosome 10, which lacks any *OsFKBP* genes, and *OsFKBP65d*, which is unanchored to any chromosome, the other 29 *OsFKBP* genes are unevenly distributed across the remaining 11 chromosomes. Chromosome 2 harbors the largest number of *OsFKBP* genes, with six identified; followed by chromosome 1 with five; chromosome 9 with four; and chromosome 7 with three genes. Chromosomes 3, 4, 6, and 8 each contain two *OsFKBP* genes, whereas chromosomes 5, 11, and 12 each support only one *OsFKBP* gene. Additionally, *OsFKBP62a*, *OsFKBP62b*, and *OsFKBP62c* are tightly clustered on chromosome 1, yet collinearity analysis reveals no evolutionary duplication among them.

### 3.3. Collinearity Analysis of FKBP Genes

**Figure 3:**
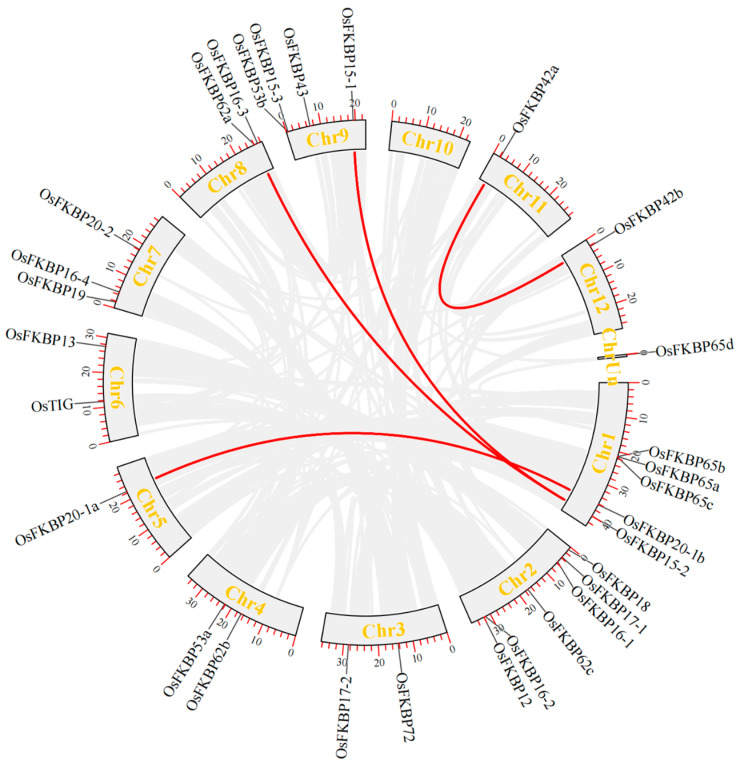
Intraspecific collinearity analysis of *OsFKBP* genes in rice. Using the One Step MCScanX Super Fast component of TBtools v2.041 software, four pairs of collinear genes (connected by red lines) were found within the rice genome.

**Figure 4:**
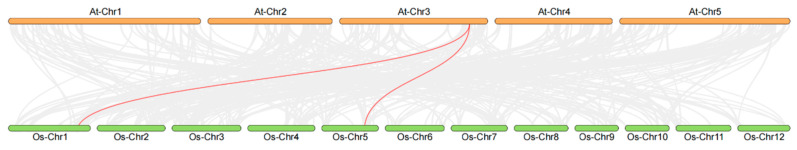
Analysis of collinearity between rice and *Arabidopsis FKBP* gene species. Use the One Step MCScanX Super Fast component of TBtools v2.041 software to view the collinearity regions in the genomes of rice and *Arabidopsis* species. Two pairs of collinear genes were found (connected by a red line).

Using the One Step MCScanX-Super Fast module of TBtools software, collinear genomic regions were identified, and collinearity maps were constructed based on positional data both within rice and between rice and *Arabidopsis* (Figure 3 and Figure 4). The analysis within species revealed four pairs of collinear genes (*OsFKBP20-1a* with *OsFKBP20-1b*, *OsFKBP42a* with *OsFKBP42b*, *OsFKBP15-1* with *OsFKBP15-2*, and *OsFKBP15-2* with *OsFKBP16-3*). This suggests that some members of the rice *OsFKBP* gene family might have originated from gene duplication events. The interspecific analysis showed that chromosomes 1 and 5 in rice are collinear with chromosome 3 in *Arabidopsis*, with two pairs of collinear genes identified between rice and *Arabidopsis* (*OsFKBP20-1a* with *AtFKBP20-1*, and *OsFKBP20-1b* with *AtFKBP20-1*). These findings imply a potential evolutionary homology between the *Arabidopsis FKBP20-1* and rice *FKBP20-1* genes.

### 3.4. Analysis of Conserved Motifs, Domains, and the Gene Structures of FKBPs

**Figure 5:**
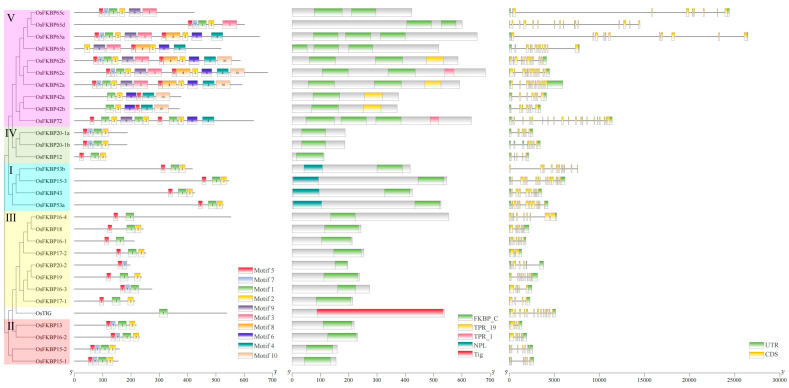
Prediction of the conservative motif (**Left**), conservative domain (**Middle**), and the gene structure (**Right**) of OsFKBPs in rice.

The conserved motifs and domains of the rice OsFKBP family were analyzed using the MEME Suite 5.5.5 online server and the NCBI-CDD online database. Based on the annotated rice genome data, a diagram of the conserved motifs, domains, and gene structures of the rice OsFKBPs was constructed (Figure 5). The analysis of conserved motifs revealed that each rice *OsFKBP* gene contains between one to ten motifs, with most containing motifs 5, 7, 1, and 2. These motifs are highly conserved components within the FKBP_C domain, and their relative positions are largely similar across the family. The analysis of conserved domains indicated that, apart from the OsTIG protein, which possesses a Tig domain, the remaining 29 rice OsFKBPs all contain the typical FKBP_C conserved domain characteristic of the FKBP gene family. The domain positions and compositions vary among different subgroups: subgroup I members have an NPL domain at the N-terminus and an FKBP_C domain at the C-terminus; subgroups II and III members have only the FKBP_C domain at the C-terminus; subgroup IV members have only the FKBP_C domain at the N-terminus; subgroup V members possess multiple FKBP_C domains, with some also having a TPR domain at the C-terminus. The gene structure analysis showed that each rice *OsFKBP* gene contains 4 to 20 coding sequences (CDS), with 27 *OsFKBP* genes having complete upstream and downstream regulatory regions (UTRs), one *OsFKBP* gene (*OsFKBP16-4*) missing the upstream UTR, and two *OsFKBP* genes (*OsFKBP53b*, *OsFKBP65*d) lacking both upstream and downstream UTRs (Figure 5).

### 3.5. Analysis of Cis-Acting Elements in FKBP Gene Promoters

**Figure 6:**
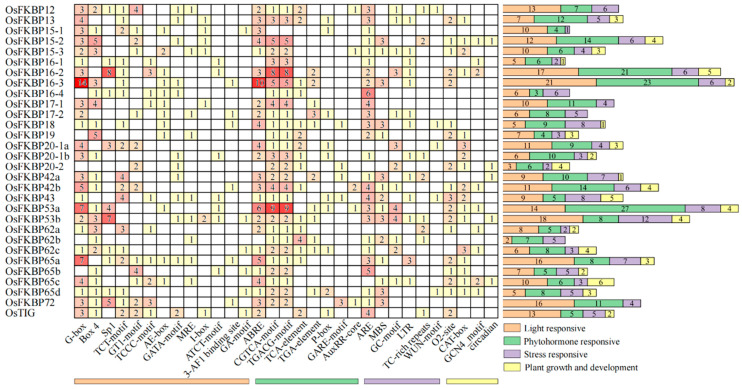
Prediction of *cis elements* in the promoter of the *OsFKBP* gene family in rice. Predictions were made through the analysis of potential *cis*-acting elements upstream of the ATG 2000 bp starting codon of the *OsFKBP* gene translation in rice using PlantCARE. Classify and arrange all predicted *cis*-acting components: Light response (orange), Phytohormone response (green), Stress response (purple), Plant growth and development (yellow). When counting the number of *cis*-acting elements for each category, the number of *cis*-acting elements for each category is marked on the right side.

*Cis*-acting elements within the promoter regions of rice *OsFKBP* genes were analyzed using the PlantCARE online platform, focusing on the 2000 bp upstream of the ATG start codon. The results reveal a substantial presence of light-responsive elements (such as G-box, Box 4, Sp1, the TCT-motif, and the GT1-motif), plant hormone-responsive elements (including abscisic acid-responsive ABRE, the methyl jasmonate-responsive CGTCA-motif, and the TGACG-motif, as well as the salicylic acid-induced TCA-element), and stress-responsive elements (such as anaerobic induction-ARE, drought response-MBS, and the hypoxia-specific induction-GC-motif) (Figure 6). These findings suggest that *OsFKBPs* likely play a crucial role in plant responses to photoperiod, hormonal signals, and environmental stressors.

In particular, light-responsive elements are the most prevalent, totaling 293, with 84 Box-4, 43 G-box, and 33 Sp1 elements. Plant hormone-responsive elements are also significant, totaling 280, including 74 ABRE, 71 CGTCA-motif, and 71 TGACG-motif elements. These data indicate that the transcription of *OsFKBP* genes may be intricately linked to photoperiods and hormonal regulation. Additionally, the genes feature 151 stress-responsive and 71 growth- and development-regulating elements, underscoring their potential regulatory complexity in plant physiology. The prediction of *cis*-acting elements on gene promoters can provide possible directions and ideas for the study of gene functions.

### 3.6. FKBP Gene Response to High-Temperature Stress

**Figure 7:**
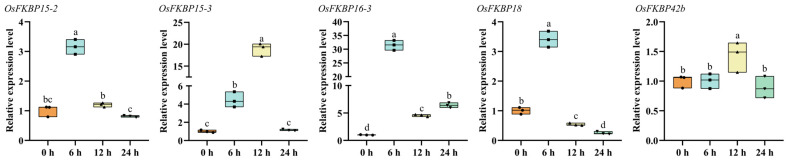
Expression analysis of some members of the *OsFKBP* genes in response to heat stress. Data are presented as mean ±SD (n = 3), and different letters indicating significant differences among these data at *p* < 0.05.

To further validate the role of the *FKBP* gene family in rice heat tolerance, the inducibility of selected *FKBP* genes through high-temperature stress was investigated. In this study, wild-type ‘Nipponbare’ rice seedlings were cultivated until day 14, after which they were exposed to high-temperature conditions at 45 °C (16 h of light and 8 h of darkness). The expression patterns of specific members of the *FKBP* gene family were monitored under these conditions. As depicted in Figure 7, post high-temperature exposure, the expression levels of *OsFKBP15-2*, *OsFKBP15-3*, *OsFKBP16-3*, *OsFKBP18*, and *OsFKBP42b* exhibited various degrees of increases. Notably, *OsFKBP15-2* and *OsFKBP1*8 demonstrated a significant up-regulation six hours into the heat treatment, which subsequently declined. Conversely, *OsFKBP15-3* and *OsFKBP42b* showed a gradual rise in expression throughout the heat treatment, peaking around 12 h before decreasing. *OsFKBP16-3* reached its maximum expression level six hours post-treatment and maintained elevated expression levels from 6 to 24 h compared to its pre-treatment baseline. Collectively, these findings indicate that *OsFKBP15-2*, *OsFKBP15-3*, *OsFKBP16-3*, *OsFKBP18*, and *OsFKBP42b* are inducible by high-temperature stress, with *OsFKBP15-3* and *OsFKBP16-3* exhibiting the most pronounced induction.

### 3.7. Analysis of FKBP Gene Responses to Drought Stress

**Figure 8:**
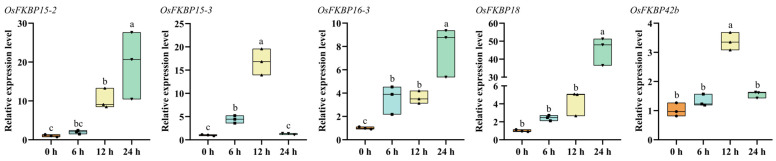
Expression analysis of some members of the *OsFKBP* genes in response to drought stress. Data are presented as mean ±SD (n = 3), and different letters indicate significant differences among these data at *p* < 0.05.

Exposure to drought stress causes significant damage to rice foliage, as documented by Li [30]. The cultivation of new drought-resistant varieties will contribute to the development of China’s rice industry [31]. In this study, wild-type ‘Nipponbare’ rice seedlings were cultivated until day 14, followed by exposure to drought conditions using a nutrient solution containing 22% PEG 6000. The expression levels of select members of the *FKBP* gene family were monitored under these stress conditions. As shown in Figure 8, compared with their levels before drought treatment, *OsFKBP15-2*, *OsFKBP15-3*, *OsFKBP16-3*, *OsFKBP18* and *OsFKBP42b* were significantly induced by drought stress, and *OsFKBP15-2*, *OsFKBP16-3* and *OsFKBP18* reached their highest levels at 24 h. The expression levels of *OsFKBP15-3* and *OsFKBP42b* reached the highest levels around 12 h after drought stress, and *OsFKBP18* had the highest level of drought induction. These FKBP family members can all be induced to be expresses by drought stress, and *OsFKBP15-2*, *OsFKBP15-3*, and *OsFKBP18* have the most drastic changes in their expression levels, which may play an important role in the rice response to drought stress.

## 4. Discussion

Previous research has demonstrated that the *FKBP* gene family is integral to both plant growth and development as well as their stress resistance mechanisms [32]. Recent advancements have led to the isolation and characterization of numerous FKBPs, revealing critical yet redundant functions within this family. The primary roles of the FKBD structural domain, however, remain elusive [31]. Currently, investigations into the *FKBP* gene family are predominantly conducted in *A. thaliana*, with significant gaps in our understanding concerning rice.

In this study, bioinformatic tools were employed to identify 30 members of the OsFKBP family from the rice genome, a slight increase from the 29 members reported by Yu, which were based on earlier research by Gollan [32,33]. Our findings suggest that this discrepancy may stem from the assembly of newly discovered genes within the rice gene database, a conclusion supported by rigorous validation. Moreover, varying strategies in gene identification often result in such discrepancies among studies, as evidenced by Suri and Ge, who identified 71 and 64 members of the wheat FKBP family, respectively [16,34]. Following their identification, these OsFKBPs were systematically named from OsTIG, and OsFKBP12 to OsFKBP72, based on their homology with the AtFKBPs from *Arabidopsis*. Phylogenetic analyses categorized the proteins, except for the distantly related TIG protein, into five distinct subgroups across all chromosomes except chromosome 10. Notably, the distribution of these genes is highly heterogeneous.

Our analysis of conserved sequences and structural domains confirms the high level of conservation observed in both sequence and structure, aligning with findings from earlier studies [4,5,6]. The *FKBP* family has been implicated in numerous stress resistance pathways in plants [16,17]. Our promoter analysis identified an abundance of stress-responsive regulatory elements, with a total of 151 elements across the 30 *FKBP* family members. This is consistent with previous studies [35,36]. Then, in order to verify this result, we selected some family members containing regulatory elements of drought stress and high-temperature stress according to the prediction results of the *cis*-acting elements, and used quantitative RT-PCR to verify whether the expression of these family members was induced by relevant stress (Figure 7 and Figure 8). It was found that the expression levels of these members were indeed up-regulated to varying degrees within 24 h after being subjected to relevant stress, and the expression levels of some members were extremely significant, which suggested that *FKBP* family members played an important role in the process of the stress resistance of rice. However, there are few studies on the function and mechanism of these *OsFKBPs*. In addition, we also predicted that the promoters of the *FKBP* family contain a large number of photo-responsive elements and plant hormone-responsive elements, which indicates that the transcription of OsFKBP genes may be related to photoperiod and plant hormones. Liu et al. reported that TWD1 (AtFKBP42)—as a described ABCB chaperone—controls stamen development through the activation of AtABCB1,19-mediated auxin transport [37]. The continual discovery and characterization of new *FKBP* family members reveal the complex interplay between these proteins and their molecular chaperones via the FKBD domain. The diverse functionalities of these chaperone proteins contribute to the complexity of FKBP functions. Furthermore, the molecular mechanisms underlying the interactions between FKBPs and their substrates are not yet fully understood and warrant deeper investigation [33].

## 5. Conclusions

In this study, 30 *FKBP*-encoding genes were systematically identified and characterized in rice, which were distributed on all chromosomes and segregated into five distinct subgroups with *Arabidopsis* except OsTIG and AtTIG. Twenty-nine rice OsFKBPs contain the typical FKBP_C conserved domain characteristic of the FKBP gene family, except for OsTIG. Further analysis of the promoter elements of these rice *FKBP* genes revealed a high presence of stress-responsive elements. Quantitative PCR assays under drought and heat stress conditions demonstrated that genes *OsFKBP15-2*, *OsFKBP15-3*, *OsFKBP16-3*, *OsFKBP18*, and *OsFKBP42b* are inducible by these adverse conditions. These results suggest a significant role for the rice *FKBP* gene family in stress adaptation. These findings not only enhance our understanding of the *FKBP* gene family in rice but also provide foundational insights for further detailed studies into their biological roles and mechanisms.

## Data Availability

No new data were created or analyzed in this study. Data sharing is not applicable to this article.

## Supplementary Materials

The following supporting information can be downloaded at: https://www.mdpi.com/article/10.3390/genes15070902/s1. Table S1. Basic information on members of the *FKBP* gene family in rice; Table S2. Primer sequence used in RT-PCR.

## References

[1] Shige Z. (2008). Progress and clinical application evaluation of immunosuppressants. Eval. Anal. Drug-Use Hosp. China.

[2] Schreiber S.L. (1991). Chemistry and biology of the immunophilins and their immunosuppressive ligands. Science.

[3] Fruman D.A., Burakoff S.J., Bierer B.E. (1994). Immunophilins in protein folding and immunosuppression. FASEB J. Off. Publ. Fed. Am. Soc. Exp. Biol..

[4] Van Duyne G.D., Standaert R.F., Karplus P.A., Schreiber S.L., Clardy J. (1993). Atomic structures of the human immunophilin FKBP-12 complexes with FK506 and rapamycin. J. Mol. Biol..

[5] Patterson C.E., Gao J., Rooney A.P., Davis E.C. (2002). Genomic organization of mouse and human 65 kDa FK506-binding protein genes and evolution of the FKBP multigene family. Genomics.

[6] Somarelli J.A., Lee S.Y., Skolnick J., Herrera R.J. (2008). Structure-based classification of 45 FK506-binding proteins. Proteins.

[7] Kurek I., Aviezer K., Erel N., Herman E., Breiman A. (1999). The wheat peptidyl prolyl cis-trans-isomerase FKBP77 is heat induced and developmentally regulated. Plant Physiol..

[8] Aviezer-Hagai K., Skovorodnikova J., Galigniana M., Farchi-Pisanty O., Maayan E., Bocovza S., Efrat Y., von Koskull-Döring P., Ohad N., Breiman A. (2007). Arabidopsis immunophilins ROF1 (AtFKBP62) and ROF2 (AtFKBP65) exhibit tissue specificity, are heat-stress induced, and bind HSP90. Plant Mol. Biol..

[9] Hueros G., Rahfeld J., Salamini F., Thompson R. (1998). A maize FK506-sensitive immunophilin, mzFKBP-66, is a peptidylproline cis-trans-isomerase that interacts with calmodulin and a 36-kDa cytoplasmic protein. Planta.

[10] Wang J., Sun W., Kong X., Zhao C., Li J., Chen Y., Gao Z., Zuo K. (2002). The peptidyl-prolyl isomerases FKBP15-1 and FKBP15-2 negatively affect lateral root development by repressing the vacuolar invertase VIN2 in *Arabidopsis*. Planta.

[11] Thirumalaikumar V.P., Gorka M., Schulz K., Masclaux-Daubresse C., Sampathkumar A., Skirycz A., Vierstra R.D., Balazadeh S. (2021). Selective autophagy regulates heat stress memory in *Arabidopsis* by NBR1-mediated targeting of HSP90.1 and ROF1. Autophagy.

[12] Meiri D., Tazat K., Cohen-Peer R., Farchi-Pisanty O., Aviezer-Hagai K., Avni A., Breiman A. (2010). Involvement of Arabidopsis ROF2 (FKBP65) in thermotolerance. Plant Mol. Biol..

[13] Wu G., Otegui M.S., Spalding E.P. (2010). The ER-localized TWD1 immunophilin is necessary for localization of multidrug resistance-like proteins required for polar auxin transport in *Arabidopsis* roots. Plant Cell.

[14] Ahn J.C., Kim D.W., You Y.N., Seok M.S., Park J.M., Hwang H., Kim B.G., Luan S., Park H.S., Cho H.S. (2010). Classification of rice (*Oryza sativa* L. *Japonica* nipponbare) immunophilins (FKBPs, CYPs) and expression patterns under water stress. BMC Plant Biol..

[15] Nigam N., Singh A., Sahi C., Chandramouli A., Grover A. (2008). SUMO-conjugating enzyme (Sce) and FK506-binding protein (FKBP) encoding rice (*Oryza sativa* L.) genes: Genome-wide analysis, expression studies and evidence for their involvement in abiotic stress response. Mol. Genet. Genom..

[16] Suri A., Singh H., Kaur K., Kaachra A., Singh P. (2022). Genome-wide characterization of FK506-binding proteins, parvulins and phospho-tyrosyl phosphatase activators in wheat and their regulation by heat stress. Front. Plant Sci..

[17] Yang D., Li Y., Zhu M., Cui R., Gao J., Shu Y., Lu X., Zhang H., Zhang K. (2023). Genome-Wide Identification and Expression Analysis of the Cucumber FKBP Gene Family in Response to Abiotic and Biotic Stresses. Genes.

[18] Zhang N., Feng S., Tian Y., Zhuang L., Cha G., Duan S., Li H., Nong X., Zhang Z., Tu X. (2023). Identification, characterization and spatiotemporal expression analysis of the FKBP family genes in *Locusta migratoria*. Sci. Rep..

[19] Paysan-Lafosse T., Blum M., Chuguransky S., Grego T., Pinto B.L., Salazar G.A., Bileschi M.L., Bork P., Bridge A., Colwell L. (2023). InterPro in 2022. Nucleic Acids Res..

[20] Finn R.D., Clements J., Eddy S.R. (2011). HMMER web server: Interactive sequence similarity searching. Nucleic Acids Res..

[21] Marchler-Bauer A., Bryant S.H. (2004). CD-Search: Protein domain annotations on the fly. Nucleic Acids Res..

[22] Li L., Zhang C., Huang J., Liu Q., Wei H., Wang H., Liu G., Gu L., Yu S. (2021). Genomic analyses reveal the genetic basis of early maturity and identification of loci and candidate genes in upland cotton (*Gossypium hirsutum* L.). Plant Biotechnol. J..

[23] Zhan Y., Wu T., Zhao X., Wang J., Guo S., Chen S., Qu S., Zheng Z. (2023). Genome-wide identification and expression of monoacylglycerol lipase (MAGL) gene family in peanut (*Arachis hypogaea* L.) and functional analysis of AhMGATs in neutral lipid metabolism. Int. J. Biol. Macromol..

[24] Chen C., Chen H., Zhang Y., Thomas H.R., Frank M.H., He Y., Xia R. (2020). TBtools: An integrative toolkit developed for interactive analyses of big biological data. Mol. Plant.

[25] Bailey T.L., Johnson J., Grant C.E., Noble W.S. (2015). The MEME Suite. Nucleic Acids Res..

[26] Lescot M., Déhais P., Thijs G., Marchal K., Moreau Y., Van de Peer Y., Rouzé P., Rombauts S. (2002). PlantCARE, a database of plant cis-acting regulatory elements and a portal to tools for in silico analysis of promoter sequences. Nucleic Acids Res..

[27] Wang Y., Huan Q., Li K., Qian W. (2021). Single-cell transcriptome atlas of the leaf and root of rice seedlings. J. Genet. Genom..

[28] Zhao J., Meng X., Zhang Z., Wang M., Nie F., Liu Q. (2023). *OsLPR5* Encoding Ferroxidase Positively Regulates the Tolerance to Salt Stress in Rice. Int. J. Mol. Sci..

[29] Zhao J., Liu X., Wang M., Xie L., Wu Z., Yu J., Wang Y., Zhang Z., Jia Y., Liu Q. (2022). The miR528-D3 Module Regulates Plant Height in Rice by Modulating the Gibberellin and Abscisic Acid Metabolisms. Rice.

[30] Li H., Wang Y., Xiao J., Xu K. (2015). Reduced photosynthetic dark reaction triggered by ABA application increases intercellular CO_2_ concentration, generates H_2_O_2_ and promotes closure of stomata in ginger leaves. Environ. Exp. Bot..

[31] Sun X., Xiong H., Jiang C., Zhang D., Yang Z., Huang Y., Li Z. (2022). Natural variation of *DROT1* confers drought adaptation in upland rice. Nat. Commun..

[32] Gollan P.J., Bhave M. (2010). Genome-wide analysis of genes encoding FK506-binding proteins in rice. Plant Mol. Biol..

[33] Yanli Y., Yanjiao L., Kaiyuan P., Fajun Z., Sun Q., Wencai L., Zhaodong M. (2014). The structure and biological function of the *FKBP* gene family in plants. Hereditas.

[34] Ge Q., Peng P., Cheng M., Meng Y., Cao Y., Zhang S., Long Y., Li G., Kang G. (2022). Genome-Wide Identification and Analysis of FKBP Gene Family in Wheat (*Triticum asetivum*). Int. J. Mol. Sci..

[35] Luan S., Kudla J., Gruissem W., Schreiber S.L. (1996). Molecular characterization of a FKBP-type immunophilin from higher plants. Proc. Natl. Acad. Sci. USA.

[36] He Z., Li L., Luan S. (2004). Immunophilins and parvulins. Superfamily Pept. Prolyl Isomerases Arabidopsis. Plant Physiol..

[37] Liu J., Ghelli R., Cardarelli M., Geisler M., Bartlett M. (2022). Arabidopsis TWISTED DWARF1 regulates stamen elongation by differential activation of ABCB1,19-mediated auxin transport. J. Exp. Bot..

