# Supplementary material for: Genome-Wide Identification and Bioinformatics Analysis of the FK506 Binding Protein Family in Rice
Source: Genes (Basel). 2024 Jul 10;15(7):902. doi: 10.3390/genes15070902 (PMC11276075; doi:10.3390/genes15070902)
Supplement: Supplementary file 1 [file genes-15-00902-s001.zip › genes-3061023-supplementary.pdf]

Table S1 Basic information on members of the FKBP gene family in Arabidopsis and rice

| FKBP in Arabidopsis | | FKBP in rice | |
|---|---|---|---|
| Gene ID | Gene Name | Gene ID | Gene Name |
| AT5G64350 | *AtFKBP12* | LOC_Os02g52290.1 | *OsFKBP12* |
| AT5G45680 | *AtFKBP13* | LOC_Os06g45340.1 | *OsFKBP13* |
| AT3G25220 | *AtFKBP15-1* | LOC_Os09g32526.1 | *OsFKBP15-1* |
| AT5G48580 | *AtFKBP15-2* | LOC_Os01g68710.2 | *OsFKBP15-2* |
| AT5G05420 | *AtFKBP15-3* | LOC_Os09g01650.1 | *OsFKBP15-3* |
| AT4G26555 | *AtFKBP16-1* | LOC_Os02g10590.1 | *OsFKBP16-1* |
| AT4G39710 | *AtFKBP16-2* | LOC_Os02g51570.1 | *OsFKBP16-2* |
| AT2G43560 | *AtFKBP16-3* | LOC_Os08g42850.4 | *OsFKBP16-3* |
| AT3G10060 | *AtFKBP16-4* | LOC_Os07g09040.1 | *OsFKBP16-4* |
| AT4G19830 | *AtFKBP17-1* | LOC_Os02g07220.1 | *OsFKBP17-1* |
| AT1G18170 | *AtFKBP17-2* | LOC_Os03g50080.1 | *OsFKBP17-2* |
| AT1G73655 | *AtFKBP17-3* | LOC_Os02g02550.1 | *OsFKBP18* |
| AT1G20810 | *AtFKBP18* | LOC_Os07g04160.2 | *OsFKBP19* |
| AT5G13410 | *AtFKBP19* | LOC_Os05g38370.1 | *OsFKBP20-1a* |
| AT3G55520 | *AtFKBP20-1* | LOC_Os01g62610.1 | *OsFKBP20-1b* |
| AT3G60370 | *AtFKBP20-2* | LOC_Os07g30800.1 | *OsFKBP20-2* |
| AT3G21640 | *AtFKBP42* | LOC_Os11g05090.1 | *OsFKBP42a* |
| AT3G12340 | *AtFKBP43* | LOC_Os12g05090.1 | *OsFKBP42b* |
| AT4G25340 | *AtFKBP53* | LOC_Os09g12270.1 | *OsFKBP43* |
| AT3G25230 | *AtFKBP62* | LOC_Os04g36890.1 | *OsFKBP53a* |
| AT5G48570 | *AtFKBP65* | LOC_Os09g01670.1 | *OsFKBP53b* |
| AT3G54010 | *AtFKBP72* | LOC_Os08g41390.1 | *OsFKBP62a* |
| AT5G55220 | *AtTIG* | LOC_Os04g28420.1 | *OsFKBP62b* |
| | | LOC_Os02g28980.1 | *OsFKBP62c* |
| | | LOC_Os01g38229.1 | *OsFKBP65a* |
| | | LOC_Os01g38180.1 | *OsFKBP65b* |
| | | LOC_Os01g38359.1 | *OsFKBP65c* |
| | | ChrUn.fgenesh.mRNA.20 | *OsFKBP65d* |
| | | LOC_Os03g25140.1 | *OsFKBP72* |
| | | LOC_Os06g20320.1 | *OsTIG* |

Table S2 Primer sequence used in RT-PCR

| Primers | Primer sequence |
|---|---|
| RT-FKBP15-2-F | CGGCCCTCATCCTGACCG |
| RT-FKBP15-2-R | CTCCGGACTTCTTGGCCGAA |
| RT-FKBP15-3-F | GTAGGAAGTACTGGCTTCAATAAAAAGC |
| RT-FKBP15-3-R | CTAGTATCTCCTCTATCACTGGTGCC |
| RT-FKBP16-3-F | GCCTGCGAGAAGGACCTCG |
| RT-FKBP16-3-R | CAGACTCTGTAGTCACCATGGGC |
| RT-FKBP18-F | GCTGGAGGACTACGTCACTTCAC |
| RT-FKBP18-R | CAAGATCATAGTACTTCAGGCCATCAG |
| RT-FKBP42b-F | TTGAAGCAGCAGACAGGAGG |
| RT-FKBP42b-R | TGCCTGTTTTCCATATTTATTTTGC |
| Actin-F | CTCGCCGACTACAACATCCA |
| Actin-R | TCTTGGGCTTGGTGTACGTCTT |
